# Dengue Outbreaks in High-Income Area, Kaohsiung City, Taiwan, 2003–2009

**DOI:** 10.3201/eid1810.111929

**Published:** 2012-10

**Authors:** Chia-Hsien Lin, Karin L. Schiøler, Martin R. Jepsen, Chi-Kung Ho, Shu-Hua Li, Flemming Konradsen

**Affiliations:** University of Copenhagen, Copenhagen, Denmark (C.-H. Lin, K.L. Schiøler, M.R. Jepsen, F. Konradsen);; National Taiwan University, Taipei, Taiwan (C.-H. Lin);; Kaohsiung City Government, Kaohsiung City, Taiwan (C.-K. Ho, S.-H. Li);; Kaohsiung Medical University, Kaohsiung City (C.-K. Ho);; and National Sun Yat-Sen University, Kaohsiung City (S.-H. Li)

**Keywords:** dengue, dengue virus, Taiwan, epidemiology, demography, Geographic Information System, urban health, socioeconomic factors, clustering, elderly, viruses, outbreak, vector-borne infections

## Abstract

Kaohsiung City, a modern metropolis of 1.5 million persons, has been the focus of dengue virus activity in Taiwan for several decades. The aim of this study was to provide a temporal and spatial description of dengue virus epidemiology in Kaohsiung City by using data for all laboratory-confirmed dengue cases during 2003–2009. We investigated age- and sex-dependent incidence rates and the spatiotemporal patterns of all cases confirmed through passive or active surveillance. Elderly persons were at particularly high risk for dengue virus–related sickness and death. Of all confirmed cases, ≈75% were detected through passive surveillance activities; case-patients detected through active surveillance included immediate family members, neighbors, and colleagues of confirmed case-patients. Changing patterns of case clustering could be due to the effect of unmeasured environmental and demographic factors.

## Abstract 2

Cases distribute in a clustered pattern, and elderly persons have the highest risk for illness and death.

Dengue virus disease or dengue virus–like disease has circulated in southern Taiwan since the late nineteenth century (*1*); transmission initially occurred as intermittent epidemics with intervals of several years to decades (*2*–*6*). However, for the past decade, dengue virus epidemics have occurred annually in Taiwan, and the main focus of activity has been in Kaohsiung City, a modern metropolis of 1.5 million persons. Kaohsiung City was the epicenter of the 2002 dengue virus epidemic, which with 2,820 confirmed cases and several hundred cases of dengue hemorrhagic fever (DHF), was one of the largest ever recorded in Taiwan (*3*). During 2002–2011, Kaohsiung City has had annual outbreaks of variable scales, resulting in ≈6,800 confirmed cases (*3*).

Cocirculation of >2 of the 4 dengue virus serotypes (DENV-1–4) has been reported in Kaohsiung City, and the molecular characteristics of the serotypes have been well documented for several epidemics, indicating the possible origin and transmission dynamics of the causative strains (*2*–*9*). The spatiotemporal patterns of disease transmission during the 2002 DENV-2 epidemic also have been investigated, and findings indicate several possible mechanisms by which the virus might have dispersed after being introduced into the population (*10*,*11*). Furthermore, Lin et al. (*12*) examined the relationship between disease-related illness and death and the distribution of primary and secondary infections for dengue virus cases reported across Taiwan during 2002–2007.

We provide a detailed description of the epidemiology of dengue virus infection in Kaohsiung City during 2003–2009; the description is based on routine disease and vector surveillance data provided by the Department of Health, Kaohsiung City Government. The temporal case distribution is compared with available climate data and the index of peridomestic adult vectors, *Aedes aegypti* and *Ae. albopictus* mosquitoes, and case characteristics are examined across the age and sex of patients and across the surveillance method (active vs. passive). In addition, the study provides an assessment of spatiotemporal case clusters, identifying possible hot spots for dengue virus transmission in Kaohsiung City during the 7-year study period.

## Materials and Methods

### Study Area

**Figure 1:**
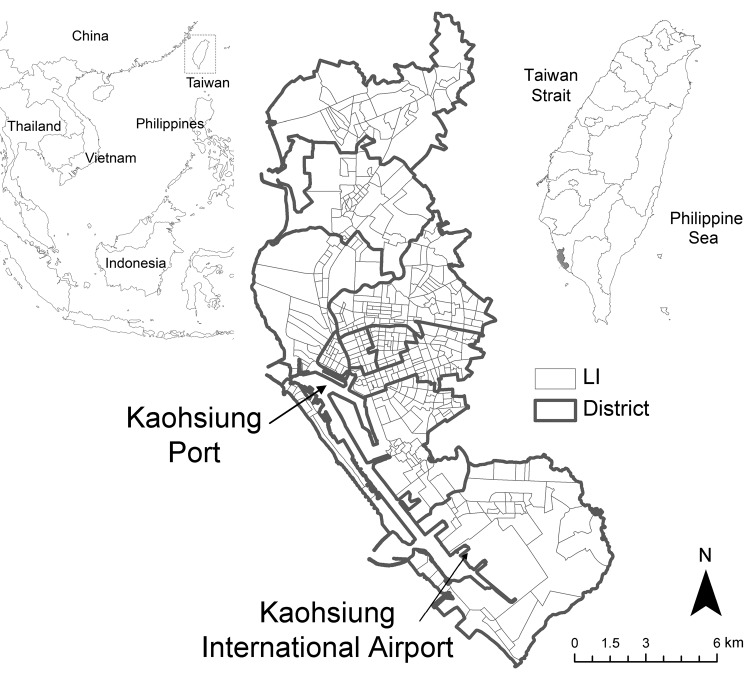
Kaohsiung City, Taiwan (22°38′N, 120°16′E), indicating the 11 districts and 463 administrative units (Li) of the city and the main entry points for international travel and commerce. Insets show location of Taiwan in Southeast Asia (box) and of Kaohsiung City in Taiwan (gray shading).

**Figure 2:**
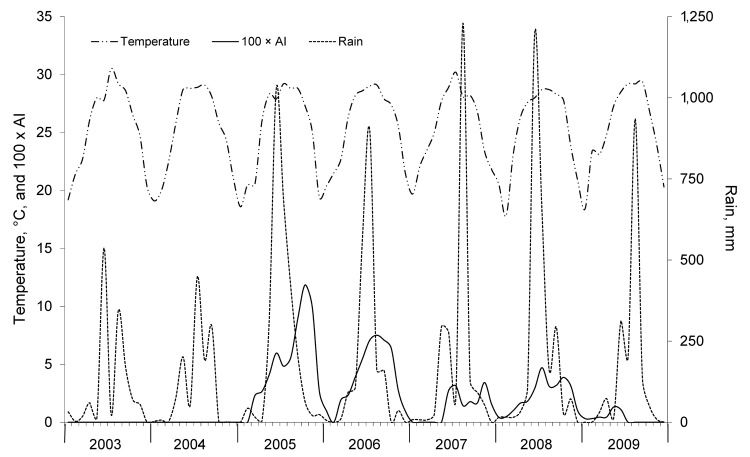
Monthly average temperature, rainfall, and adult index (AI) for *Aedes aegypti* and *Ae. albopictus* mosquitoes, Kaohsiung City, Taiwan, 2003–2009. AI was calculated as number of adult female mosquitoes captured per number of inspected premises.

Kaohsiung City, located on the southwestern coast of Taiwan (22°38′N, 120°16′E), has a tropical monsoon climate (*13*) (Figure 1). During the study period, January 2003–December 2009, the highest annual temperatures were during June–August (monthly mean temperature range 28.7°C–30.5°C), and the lowest temperatures were recorded during January and February (mean temperature 18.4°C and 20.4°C, respectively). The highest monthly accumulative precipitations occurred during June–August (range 901.5–1,229.3 mm), whereas there was almost no precipitation during November–February (*14*) (Figure 2).

Kaohsiung City is a major port and industrial metropolis and the most densely populated urban center in Taiwan (1.5 million persons within a total area of 150 km^2^). The city is divided into 11 districts; a Li is the smallest administrative unit within these districts. The overall number of Lis decreased from 463 to 459 in 2006. On average, the population density of a Li is 150–65,000 persons/km^2^ (Figure 1). Piped water is available for 99% of the city households, and household waste is removed daily throughout the city by the Environmental Protection Bureau, Kaohsiung City Government. The city has an international airport, which is a major access point for tourists and foreign workers, many of whom are employed in the commercial port and the industrial zones of the city. Most of the 15,000 foreign workers who arrive in the city each year are citizens from neighboring countries, such as the Philippines, Indonesia, Vietnam, and Thailand (*15*).

### Dengue Surveillance and Laboratory Diagnosis

The disease surveillance system in use during the study period was introduced under the Communicable Disease Prevention Act in 1999 by the Taiwan Center for Disease Control (Taiwan CDC). The system ensured reporting and laboratory confirmation of all suspected cases of dengue virus infection identified through passive or active surveillance activities, using the probable case definition of the World Health Organization (WHO) (*16*). Passive surveillance involved mandatory reporting of probable dengue virus infection cases to Taiwan CDC within 24 hours after a patient sought medical assistance at any of the city’s 1,747 health facilities, including public health centers, general practitioner clinics, and public and private hospitals. Passive surveillance activities also involved school-based reporting of febrile students and self-reporting to health authorities by citizens with probable dengue virus infection (*3*). Active surveillance included fever checkpoints at the airport and screening by the district public health nurse of immediate contacts (e.g., family members, colleagues, and neighbors) of persons with confirmed dengue virus infection (*3*,*17*). Basic patient information was collected and blood samples were obtained from all suspected case-patients and sent to the Fifth Branch Office of Taiwan CDC in Kaohsiung City.

A patient was confirmed to have dengue virus infection if 1) dengue virus RNA was detected in a serum sample by real-time reverse transcription PCR, 2) dengue virus–specific antibodies were detected in single serum samples by IgM- or IgG-capture ELISA, or 3) a >4-fold increase in IgG ELISA titers was detected in paired acute- and convalescent-phase serum samples (*3*). In August 2008, detection of dengue virus nonstructural protein 1 by use of a rapid diagnostic test (Platelia Dengue NS1 Ag assay; Bio-Rad, Marnes la Coquette, France) was included as a fourth diagnostic method (*3*). DHF, including dengue shock syndrome (DSS), was distinguished from dengue fever by the presence of 1) hemorrhagic tendencies, including a positive tourniquet test result and bleeding from the mucosa, gastrointestinal tract (hematemesis, hematuria, or melena) or other locations; 2) thrombocytopenia (<100,000 cells/mm^3^); or 3) plasma leakage (*3*). The severity of DHF was not further classified. All laboratory tests and most of the incurred medical expenses were covered by the National Health Insurance.

### Patient Data

During the study period, January 2003–December 2009, patient data for all laboratory-confirmed cases were provided by the Department of Health, Kaohsiung City Government. The data included the registered home address, sex, date of birth, date of manifestation onset, surveillance methods (active or passive), and reported clinical manifestations (fever, anorexia, headache, arthralgia, rash, myalgia, thirst, diarrhea, nausea, pruritus, vomiting, retro-orbital pain, and hemorrhagic manifestations).

### Vector Index

Vector surveillance activities by the Department of Health, Kaohsiung City Government, were initiated in 2005 by using specially trained personnel. The Li was used as the surveying unit in which 50–100 households were randomly selected for inspection of *Ae. aegypti* and *Ae. albopictus* mosquito infestation (*3*). Adult *Aedes* mosquitoes were captured indoors and outdoors with hand-nets at 8:30–11:30 am or 1:30–4:30 pm (*3*). Capture activities were completed for all rooms, including the basement, within a maximum of 10 minutes for each inspected premise. The adult index was calculated as the number of adult female mosquitoes captured divided by number of inspected premises.

### Epidemiologic Analysis

Incidence rates and clinical manifestations were calculated for age-specific groups and sex by using the year-end population data for each study year as the denominator. The *z* test was applied for incidence rate comparison. The 2-sample *t* test was used for the comparison of the average number of clinical manifestations in patients detected in the passive versus the active surveillance system. The threshold of statistical significance was 0.05.

### Spatial Analysis

Spatial patterns of dengue incidence in each Li were assessed by use of global and local indices. The global spatial pattern was measured by using Moran’s I, an index of spatial autocorrelation coefficient, yielding only 1 summary statistic for the whole study area. The theoretical range of Moran’s I was from −1 to 1; the value around 0 provided the indication of spatial random distribution. Higher positive values implied a stronger clustering pattern, and lower negative values represented a stronger dispersion tendency (*18*). We determined partial autocorrelation by using global statistics and actual cluster location by using the local indicator of spatial association (LISA). Anselin’s LISA provided the local version of Moran’s I, used here to compare mean incidence rates for each Li and its neighboring Lis (*19*). The mapped LISA results indicated how spatial autocorrelation varied over the study region according to 5 categories: 1) hot spot, denoting a high-incidence Li surrounded by high-incidence Lis; 2) high-value outlier, denoting a high-incidence Li surrounded by low-incidence Lis; 3) low-value outlier, denoting a low-incidence Li surrounded by high-incidence Lis; 4) cold spot, denoting a low-incidence Li surrounded by low-incidence Lis; 5) not significant, denoting no spatial autocorrelation presented.

All epidemiologic and temporal analyses were performed by using Excel 2002 (Microsoft, Redmond, WA, USA) and R-2.7.2 for Windows (http://cran.r-project.org/bin/windows/base/old/2.7.2/). Spatial analyses were done by using ArcGIS 9.2 (ESRI, Redlands, California, USA).

## Results

During January 2003–December 2009, Taiwan CDC recorded 2,087 laboratory-confirmed cases of dengue virus infection in Kaohsiung City. The cases were detected by passive and active surveillance activities. Of the confirmed cases, 98.7% (2,060) were classified as dengue fever and 1.3% (27) as DHF/DSS. The 7-year fatality rate for patients with DHF/DSS was 25.9% (7/27).

### Temporal Case Distribution

**Figure 3:**
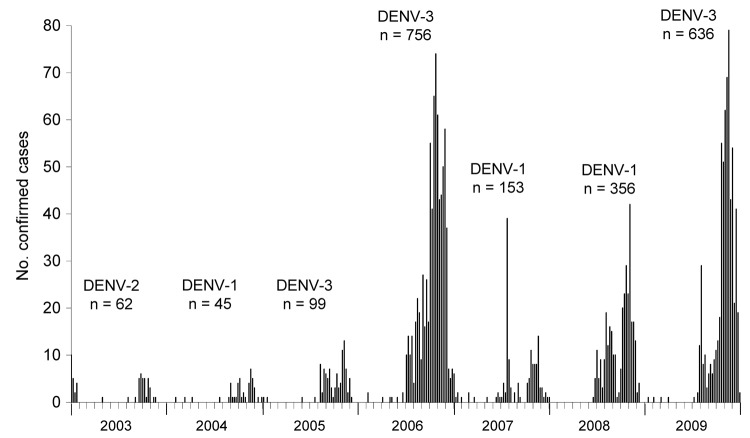
Epidemic curve of confirmed cases of dengue virus (DENV) infection (N = 2,087), by week of onset, Kaohsiung City, Taiwan, 2003–2009. Predominant serotypes (DENV-1–3) and numbers of confirmed cases are shown.

Most (96.9%) of the confirmed cases of dengue virus infection were recorded during epidemics occurring during July–December of each year. The interannual variations in outbreak scale were considerable, ranging from 45 confirmed patients in 2004 to 766 in 2006. A dominant serotype was evident during each epidemic, representing >80.0% of cases confirmed by virus detection (real-time reverse transcription PCR) in a given year (Figure 3).

The annual onset of epidemic activity generally coincided with the peak in monsoon rainfall and temperature levels (Figure 2 and Figure 3). The epidemic peaked within 1–3 months after the onset of the epidemic, and all activity ceased at the end of the monsoon season. Vector data for 2005–2009 showed that the peak of the adult mosquito population followed the peak of the monsoon rainfall, with a lag period of 1–2 months that corresponded to disease activity (Figure 2 and Figure 3).

### Characteristics of Case-Patients

#### Age

The median age of patients with confirmed dengue virus infection was 46 years (range 4 months–95 years). The average age-specific incidence rate was lowest among persons <5 years of age (4.5/100,000 persons) and increased steadily by age group (12.0, 14.3, 15.3, 17.9, and 25.3/100,000 persons for case-patients 5–14, 15–24, 25–34, 35–44, and 45–54 years of age, respectively) until peaking in persons 55–64 years of age (37.9/100,000 persons) (Table 1). The pattern of observed age-specific incidence rates was uniform across all epidemics during the study period (data not shown).

**Table 1:** Age- and sex-specific incidence rates of confirmed cases of dengue virus infection, Kaohsiung City, Taiwan, 2003–2009*

Age group, y, sex	No. cases	Incidence rate/100,000 persons	p value†	No. DHF cases	No. fatal cases	Fatality rate, %
0–4						
M	14	5.7	0.184	2	0	0
F	7	3.1		0	0	0
All	21	4.5		2	0	0
5–14						
M	90	12.7	0.497	0	0	0
F	74	11.3		1	0	0
All	164	12.0		1	0	0
15–24						
M	134	17.1	0.002‡	2	0	0
F	82	11.1		2	0	0
All	216	14.3		4	0	0
25–34						
M	144	15.9	0.582	0	0	0
F	135	14.7		0	0	0
All	279	15.3		0	0	0
35–44						
M	157	18.2	0.968	1	0	0
F	161	17.7		0	0	0
All	318	17.9		1	0	0
45–54						
M	189	23.4	0.097	0	0	0
F	234	27.1		0	0	0
All	423	25.3		0	0	0
55–64						
M	167	35.0	0.142	1	0	0
F	210	41.1		4	1	25.0
All	377	37.9		5	1	20.0
65–74						
M	96	35.0	0.352	5	2	40.0
F	118	39.4		4	1	25.0
All	214	37.3		9	3	33.3
>74						
M	44	21.9	0.418	5	3	60.0
F	31	18.1		0	0	0
All	75	20.2		5	3	60.0
Total						
M	1,035	19.9	0.624	16	5	31.3
F	1,052	19.4		11	2	18.2
All	2,087	19.6		27	7	25.9

*DHF, dengue hemorrhagic fever. †Difference in incidence between male and female population for the age group given. ‡Level of significance p<0.05.

Among 27 patients with confirmed cases of DHF, fatality was highest among those >74 years of age (3/5, 60.0%) followed by those 65–74 years of age (3/9, 33.3%) and 55–64 years of age (1/5, 20.0%). No fatalities occurred among other age groups (Table 1).

#### Sex

For both sexes, persons 55–64 and 65–74 years of age had the highest and second highest incidence rates of confirmed dengue virus infection (Table 1). Overall, the incidence rate for the female population was slightly, but not significantly, higher than that for the male population (19.9 vs. 19.4/ 100,000 persons; p = 0.624). Among persons 15–24 years of age, the incidence rate for the male population was significantly higher than that for the female population (17.1 vs. 11.1/100,000 persons; p = 0.002).

### Case Detection by Active and Passive Surveillance

**Figure 4:**
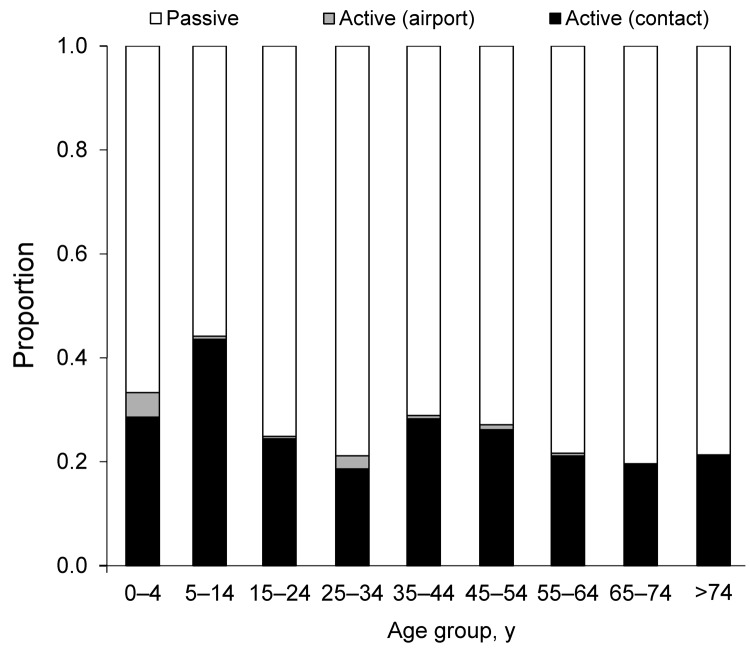
Age-specific distribution of case-patients with confirmed dengue virus infection (N = 2,087) detected by passive and active surveillance systems, Kaohsiung City, Taiwan, 2003–2009. Cases detected through passive surveillance were suspected dengue virus infections reported by health care facilities to Taiwan Center for Disease Control; cases detected through active surveillance were reported from airport screenings and by community contacts of case-patients.

The active surveillance system detected 538 (25.8%) of the confirmed cases during 2003–2009. Of these cases, 520 (96.7%) were in household members, neighbors, or colleagues of confirmed case-patients, and 18 were detected by the fever-screening system at the airport. The highest proportions of cases detected through active surveillance were among children 0–4 and 5–14 years of age (Figure 4).

The average number of reported disease manifestations was significantly higher among cases detected through passive than through active surveillance (n = 5.1 vs. 3.7; p<0.001), and each clinical manifestation was reported more frequently through the passive than the active system, with the exception of pruritus (Table 2). In both surveillance systems, the number of reported manifestations was lowest for persons 0–4, 65–74, and >74 years of age: average of 2.2, 1.9, and 1.2 manifestations, respectively, when detected through active surveillance (Table 3). These numbers of reported clinical manifestations are lower than the number required to meet the WHO criterion for probable dengue virus infection.

**Table 2:** Comparison of reported clinical manifestations in persons with confirmed dengue virus infection (N = 2,087) detected through passive or active surveillance, Kaohsiung City, Taiwan, 2003–2009

Manifestation	No. passive (%), n = 1,549	No. active (%), n = 538	p value*
Fever	1,509 (97.4)	392 (72.9)	<0.001
Anorexia	916 (59.1)	218 (40.5)	<0.001
Headache	829 (53.5)	224 (41.6)	<0.001
Arthralgia	765 (49.4)	166 (30.9)	<0.001
Rash	760 (49.1)	209 (38.8)	<0.001
Myalgia	754 (48.7)	175 (32.5)	<0.001
Thirst	694 (44.8)	168 (31.2)	<0.001
Diarrhea	486 (31.4)	121 (22.5)	<0.001
Nausea	447(28.9)	95 (17.7)	<0.001
Pruritus	324 (20.9)	120 (22.3)	0.4941
Vomiting	296 (19.1)	53 (9.9)	<0.001
Retro-orbital pain	182 (11.7)	43 (8.0)	<0.001
Hemorrhagic manifestations	96 (6.2)	11 (2.0)	<0.001

*Proportional difference in reporting of specific manifestation by passive and active surveillance.

**Table 3:** Age-specific frequencies of reported clinical manifestations and average reported number of manifestations for confirmed dengue cases (N = 2,087) detected through passive or active surveillance, Kaohsiung City, Taiwan, 2003–2009

Variable	Age group, y								
	0–4	5–14	15–24	25–34	35–44	45–54	55–64	65–74	>74
Passive surveillance system									
No. cases	14	92	161	220	226	310	297	172	57
Average no. manifestations	3.4	5.0	5.7	5.9	5.5	5.1	4.9	4.5	3.6
Manifestation, no.									
Fever	1.0	1.0	1.0	1.0	1.0	1.0	1.0	0.9	0.9
Anorexia	0.4	0.7	0.6	0.6	0.6	0.6	0.6	0.6	0.6
Headache	0.1	0.5	0.7	0.6	0.6	0.6	0.5	0.4	0.3
Arthralgia	0.0	0.2	0.5	0.6	0.6	0.5	0.5	0.5	0.3
Rash	0.6	0.7	0.6	0.6	0.5	0.4	0.4	0.3	0.3
Myalgia	0.1	0.3	0.5	0.6	0.6	0.5	0.5	0.4	0.3
Thirst	0.3	0.3	0.4	0.5	0.5	0.5	0.5	0.4	0.2
Diarrhea	0.3	0.3	0.3	0.3	0.3	0.3	0.3	0.3	0.3
Nausea	0.0	0.3	0.4	0.4	0.3	0.3	0.2	0.3	0.2
Pruritus	0.1	0.3	0.3	0.3	0.3	0.2	0.2	0.1	0.0
Vomiting	0.4	0.3	0.2	0.2	0.2	0.2	0.2	0.2	0.2
Retro-orbital pain	0.0	0.1	0.2	0.2	0.2	0.1	0.1	0.0	0.0
Hemorrhagic manifestations	0.0	0.0	0.1	0.1	0.0	0.1	0.1	0.1	0.1
Active surveillance system									
No. cases	7	72	55	59	92	113	80	42	18
Average no. manifestations	2.2	3.5	4.4	4.6	4.6	3.8	3.1	1.9	1.2
Manifestation, no.									
Fever	1.0	0.8	0.9	0.9	0.8	0.8	0.6	0.4	0.4
Anorexia	0.1	0.3	0.5	0.4	0.5	0.4	0.4	0.3	0.2
Headache	0.0	0.5	0.5	0.6	0.5	0.4	0.3	0.2	0.3
Arthralgia	0.0	0.2	0.2	0.4	0.5	0.4	0.3	0.2	0.2
Rash	0.4	0.5	0.5	0.5	0.4	0.4	0.3	0.1	0.0
Myalgia	0.0	0.2	0.3	0.5	0.5	0.4	0.3	0.1	0.1
Thirst	0.1	0.2	0.3	0.4	0.4	0.4	0.3	0.2	0.1
Diarrhea	0.3	0.2	0.2	0.3	0.3	0.2	0.2	0.1	0.1
Nausea	0.0	0.1	0.3	0.2	0.2	0.2	0.2	0.1	0.0
Pruritus	0.1	0.3	0.4	0.3	0.3	0.2	0.2	0.1	0.0
Vomiting	0.0	0.1	0.2	0.1	0.1	0.1	0.1	0.1	0.0
Retro-orbital pain	0.0	0.1	0.1	0.1	0.1	0.1	0.0	0.0	0.0
Hemorrhagic manifestations	0.0	0.0	0.0	0.0	0.0	0.0	2.6	0.0	0.0

### Spatial Analysis

**Figure 5:**
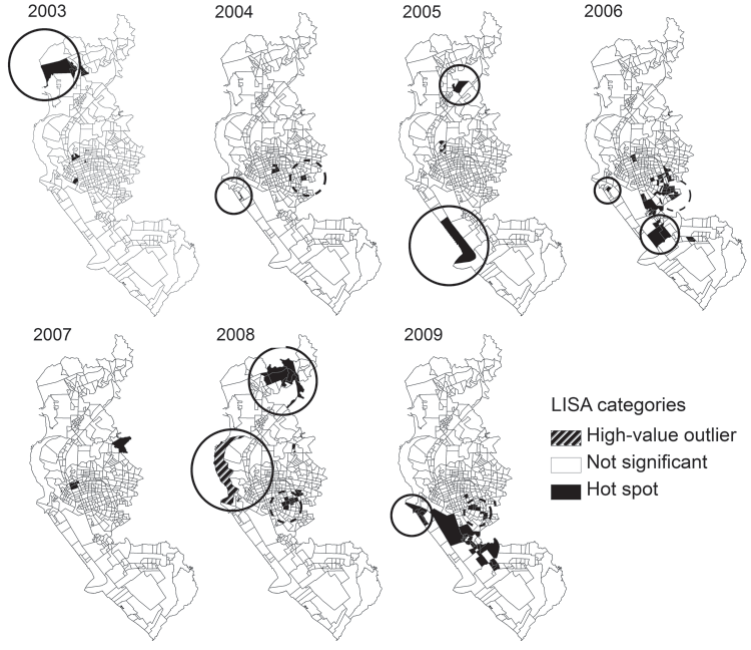
Local indicator of spatial association (LISA) cluster maps of incidence rates for dengue virus infection during each epidemic period, Kaohsiung City, Taiwan, 2003–2009. High-value outlier, high-incidence Li (smallest administrative unit within each of 11 districts in Kaohsiung City) surrounded by low-incidence Lis; not significant, 0 spatial autocorrelation presented; Hot spot, high-incidence Li surrounded by high-incidence Lis. Hot-spot Lis circled with dashed lines are those that overlap with clusters of high residential density; hot-spot or high-value outlier Lis circled with solid lines are those that overlap with clusters of low residential density.

The global level of spatial autocorrelation for the dengue virus infection incidence rates across the Lis of Kaohsiung City was significant for each epidemic (range 0.03–0.14, Moran’s I; p<0.001) (Table 4), indicating a significant positive spatial autocorrelation within the city for each epidemic year. The type and area of local clustering, as determined by LISA, were identified for each epidemic. In general, hot-spot Lis with a high incidence of infection did not overlap for consecutive years; however, certain hot spots recurred or were adjacent to other hot spots for the epidemics of 2004, 2006, 2008, and 2009. These hot spots overlapped with clusters of high residential density. Some dengue hot spots were also observed for areas of low population density throughout the study period, except for 2004 and 2007. Half of the high-value outliers were observed in low population clusters (Figure 5).

**Table 4:** Spatial autocorrelation of dengue incidence rates in Kaohsiung City, Taiwan, 2003–2009

Year	No. cases	Moran’s I*	p value†
2003	62	0.09	<0.001
2004	45	0.08	<0.001
2005	99	0.04	<0.001
2006	766	0.14	<0.001
2007	153	0.03	<0.001
2008	326	0.10	<0.001
2009	636	0.09	<0.001

*An index of spatial autocorrelation coefficient. †p<0.05 indicated that the spatial pattern was nonrandom.

## Discussion

Evidence from Kaohsiung City shows that dengue virus infection occurs in persons of all ages; however, the incidence of infection increases notably in persons of increasing age, and elderly persons are at especially high risk for DHF/DSS and death. Most case-patients detected by the active surveillance system were contacts of case-patients detected by the passive surveillance system. Changes in disease hot spots were noted between successive years of the study.

The findings in this study support previous reports of a clear correlation between precipitation, temperature, and the occurrence of dengue virus epidemics in Taiwan (*20*,*21*). For Kaohsiung City, the study shows that the annual onset of dengue virus epidemics coincides with the time of peak rainfall and temperature. Dengue virus outbreaks coincide with the seasonal increase of adult vectors in the peridomestic environment, but there seems to be no correlation between the level of vector abundance and the scale of outbreaks. The same can be concluded for the meteorologic variables assessed in this study. This observation is in line with the general understanding that the extent of dengue virus epidemics may be influenced by a variety of factors, including the level of herd immunity to the circulating serotype(s); the virulence of the circulating strain(s); and the effect of human-vector contact exerted by human behavior, specific climatic phenomena, and prevention and control operations.

All 4 dengue virus serotypes were identified in Kaohsiung City, and at least 2 serotypes cocirculated during each outbreak of the study period. DENV-1 and DENV-3 were clearly predominant during 3 outbreaks each; DENV-2 detection was limited, and DENV-4 detection was negligible. Specific information on the circulating genotypes or strains was not available for assessment. However, recent studies suggest that most dengue virus outbreaks in Taiwan can be attributed to the importation of novel dengue virus strains from neighboring Southeast Asian countries, in particular those with which substantial immigration, tourism, and trade relations are maintained (*4*,*7*,*8*).

The age distribution of confirmed dengue case-patients in Kaohsiung City was consistent throughout the study period. Children <5 years of age had the lowest disease incidence, and persons 55–64 and 65–74 years of age had the highest incidence of confirmed cases, including cases of DHF and dengue virus–related deaths.

The low incidence of dengue virus infection among the youngest age group fits well with descriptions of mild or mainly asymptomatic dengue virus infection in younger children (*22*). However, it is often suggested that the lack of vocal ability among small children plays a factor in the health-seeking behavior of their caretakers. In either case, one would expect a relatively larger group of 0- to 4-year-old children than older persons to be identified through the active surveillance system. In fact, the 0- to 4-year-old age group had the second highest proportion of cases detected through active surveillance (Figure 4); however, the overall detection rate remained far below that for all other age groups, suggesting generally lower rates of dengue virus exposure. This finding could be attributed to specific behavioral aspects of urban living in a high-income setting, such as Kaohsiung City, where, young children spend most of their day in enclosed air-conditioned environments at their home, day care center, or preschool. Hence, vector exposure may be substantially lower for young Taiwanese children than for most of their peers in other Southeast Asian countries.

The case-fatality rate of 26% for older age groups in this study is far greater than the expected average of <1% reported by WHO (*23*). However, our findings correspond with those in previous reports from Taiwan (*12*,*24*–*26*), where underlying chronic diseases more commonly observed among older persons (e.g., hypertension, chronic renal insufficiency [uremia], or diabetes mellitus) have been identified as possible risk factors for severe and fatal DHF (*27*,*28*). The association between age, the presence of underlying disease, and severe dengue virus infection and related death has also been reported from Cuba (*29*,*30*). However, these findings have been disputed by findings from Singapore, where elderly patients did not exhibit more signs or symptoms of dengue virus infection or have higher death rates despite having a greater incidence of underlying diseases (*31*). Information about the presence of underlying diseases was not available for our study.

Overall in Kaohsiung City, the incidence of dengue virus infection in the female population was slightly higher than that in the male population. This finding was not statistically significant, but it was in agreement with findings from previous studies conducted in Taiwan and contrary to findings from several other Asian countries where dengue is reported more frequently among the male population (*32*–*35*). These data suggest that the risk for exposure to dengue virus in Kaohsiung City is shared between sexes. It may also suggest that the combination of passive and active surveillance activities eliminates potential differences in health care–seeking behavior or health care access, as has been suggested for other Asian countries with lower reported rates of cases among the female population (*34*).

More than 25% of the confirmed dengue virus cases were detected through the active surveillance system. Most (96.7%) of these cases involved household members, neighbors, or colleagues of case-patients detected through the passive surveillance system. The 0- to 4-year-old and 5- to 14-year-old age groups had the highest proportion of cases (33.3% and 44.2%, respectively) detected through active surveillance, a finding in line with the lower number of reported symptoms (milder disease) and the possibly greater likelihood of being at home when the active surveillance nurse visited.

The inherent underreporting of dengue virus infections by passive surveillance, caused by mild and asymptomatic infections, is counterbalanced by the addition of the active surveillance component, although the level of impact remains unknown. High retrieval rates for convalescent-phase 2 and 3 samples (obtained for 455 [21.8%] of the 2,087 cases) ensure that all reported cases were thoroughly analyzed by molecular and serologic testing. However, the sensitivity and specificity of the surveillance and laboratory diagnostic systems and the overall cost-effectiveness of the surveillance and control program in Kaohsiung City must be assessed (*36*).

Elderly case-patients in both surveillance systems reported fewer symptoms, indicating the urgent need for improved diagnosis and treatment of severe dengue virus infection in this high-risk population. However, improved diagnosis and treatment would require better detection of cases that do not fit the currently used criteria for probable dengue virus infection. In addition, consideration would need to be given to the potential influence of underlying disease in treatment for severe dengue virus infection.

The incidence rates of dengue virus cases occurred nonrandomly throughout Kaohsiung City, implying that risk factors for dengue virus infection were spatially heterogeneous (Table 4). Hot-spot Lis were detected in different locations during consecutive years (Figure 5), although some hot spots recurred or were adjacent to other hot spots for the epidemics of 2004, 2006, 2008, and 2009. These hot spots were all shown to overlap with areas of high human residential density. Other hot spots and high-value outliers were detected in areas of low and high population density.

The ambiguous correlation between the incidence of dengue virus infections and population density was also reported by Lin and Wen for the 2002 DENV-2 epidemic in Kaohsiung City (*37*), indicating that variations in population density are insufficient for explaining spatial variations in dengue virus outbreak intensity at the local level. To understand the observed variations and to predict local occurrence of future outbreaks, it is therefore necessary to account for additional spatial factors of potential importance.

Our findings on dengue virus outbreaks and high-risk population groups suggest the need for further research on demographic parameters, such as age distribution and age-dependent behavior at the local level. Variations in socioeconomic status, housing standards, and housing density should also be investigated for a potential role in disease clustering (*38*–*40*). In addition, the distinct seasonal transmission pattern of dengue virus shown in this study suggests further research into local environmental factors and control activities that influence vector survival and availability and productivity of vector breeding sites. Refined spatial analysis combining data for all spatial factors of identified importance could help identify imminent hot spots and guide improved prevention and control efforts.
